# High resolution mapping of a cold water coral mound

**DOI:** 10.1038/s41598-018-37725-x

**Published:** 2019-01-31

**Authors:** Luis A. Conti, Aaron Lim, Andrew J. Wheeler

**Affiliations:** 1 Escola de Artes Ciências e Humanidades, Universidade de São Paulo, São Paulo, Brazil; 2 School of Biological, Earth & Environmental Sciences, University College Cork, Cork, Ireland; 3 Environmental Research Institute, University College Cork, Cork, Ireland; 4 Irish Centre for Research in Applied Geosciences/Marine & Renewable Energy Institute, University College Cork, Cork, Ireland

## Abstract

Cold-water coral (CWC) mounds are biogenic, long-lived morphostructures composed primarily by scleractinian CWC’s and hemipelagic sediments that form complex deep-sea microhabitats found globally but specifically along the European-Atlantic margin. In this work, high-resolution mapping was applied to identify individual organismal distribution and zonation across a CWC Piddington Mound within the Porcupine Seabight, Ireland Margin. Marine Object-Based Image Analysis (MOBIA) and different machine learning classification methods (decision tree, logistic regression, and deep neural network) were applied to a high-resolution (2 mm) reef-scale video mosaic and ROV-mounted multibeam data in order to provide new insights into the spatial organization of coral frameworks and environmental factors on CWC mounds. The results showed an accurate quantification of the amount of Coral Framework (14.5%; ~2% live and ~12.5% dead) and sponges (~3.5%) with heterogeneous distribution, restricted to a certain portion of the mound. This is the first object level quantification of live and dead coral framework facies and individual sponges across an entire CWC mound. This approach has application for habitat and conservation studies, provides a quantification tool for carbon budget assessments and can provide a baseline to assess CWC mound change. The approach can also be modified for application in other habitats.

## Introduction

The extent of geographical range and ecological functioning of submarine benthic habitats are still poorly understood^1–3^. It is estimated that less than 5% of the seafloor is mapped at a resolution to that of similar studies on land^4^. Given the inherent difficulties of seabed mapping, specifically in relation to acquisition and analysis of marine information, new methods have now been proposed to increase knowledge from this unmapped part of the planet^5,6^. These include the use of structure from motion derived 3D photogrammetric reconstructions of deep-water habitats and the use of multifrequency multibeam backscatter for improved interpretations of subtle seabed features^7,8^.

For cold water coral (CWC) reefs and mounds, some early mapping efforts utilised regional-scale side scan sonar or multibeam echo sounder to investigate bioconstruction morphologies and seabed processes^9–13^. Later, predictive modelling and habitat suitability modelling were employed on these habitats which outlined their distribution over large areas and pointed out the need for more local-scale studies^14–16^. More recently, photogrammetry, ROV- and AUV-mounted multibeam mapping have revealed their local-scale distribution on relatively flat areas^17^ to near-vertical canyon walls^18,19^.

Despite this potential improvement of mapping seafloor habitats with coupled “video/sonar” data, seabed optical images derived from photo/video cameras mounted on Remote Operated Vehicles (ROVs) in many cases, remain limited by exploratory survey designs^20–22^, ground truthing^23–25^ and rapid ecological assessment^26^. In particular, efforts to map deep-water, CWCs have achieved considerable development after the use of integrated multi data spatial analysis^11,27–29^.

One of the difficulties of integrating multibeam (e.g. bathymetry and backscatter) with image data is the problem of spatial scale sensitivity to facies and habitat classification. For example, combining images of different resolution tends to increase internal variability and noise within classes and therefore may decrease the classification accuracy of traditional per-pixel basis methods^30^.

The concept of Geographic Object-Based Image Analysis (GEOBIA) was proposed in order to overcome problems of noise and misclassification^31–35^ and is particularly well-suited to the analysis of very high resolution (VHR) images where the increased heterogeneity of sub-meter pixels would otherwise confuse pixel-based classifications^36^. OBIA consists of two inter-related steps: segmentation and classification. The segmentation step is based on the creation of semantically and “meaningful” objects (polygons) based on groups of neighboring pixels with similar spectral and spatial properties. The second step is object classification which consists of allocating each object to some preselected classes based on its spectral, textural, spatial and topological characteristics. OBIA classification can offer a methodological framework for machine-learning methods which takes into account multiple properties of image objects^31,37^.

Diesing^38^ adapted the term to Marine Object-Based Image Analysis (MOBIA) as the application of GEOBIA to marine data sets with the aim of mapping seafloor geomorphology, geology and habitats. The use of MOBIA, however, is still lacking in more widespread applications. There are some explanations for this, firstly, the spatial distribution of the dynamics of marine and ocean phenomena (entities and processes) do not conform to patterns established with those from terrestrial areas. Furthermore, there are no robust, established theoretical models that can be used to characterize the spatial hierarchy of marine phenomena, especially in relation to ecological interactions, which could guide the establishment of spatial units based, for example, on watershed analysis or Landscape Ecology (see^39^). Manderson^40^, in addressing this question, indicates that the basic analysis of marine environments following an ecosystem approach should always be associated with the scale of the dominant hydrodynamic process. The derived “Benthoscapes” in this context would reflect a set of spatially enclosed habitats controlled by a specific hydrodynamic process. The controlling process however could be difficult to detect by remote sensing techniques making this task difficult or subjective^41^.

Secondly, the original “object” modeling methodologies developed for terrestrial areas were primarily for optical multispectral satellite image analysis, which allows a direct and clear delimitation of spatial aggregation units. In the case of submarine environments, acoustic remote sensing (through multibeam sonars or side scan sonars, for example) does not add as much information; the variability in the acoustic signal is directly related to the type of substrate (texture and composition) which does not necessarily reflect direct ecological characteristics. As such, it can be said that much of the factors that characterize the variation within marine environments (or habitats) are “invisible” to acoustic signals.

Recent studies indicate that promising results for the characterization of “Benthoscapes” from the segmentation and application of OBIA of marine substrates at the meter-scale resolution can be achieved using optical data such as video-imaging (examples in^42,43^). In the case of optical-scale surveys, in which the variation of environments is evident such as with coral reefs, object-oriented classification has shown even more consistent results^44,45^. A pioneer study by Purser *et al*.^46^, showed that machine learning of optical-scale survey data provided accurate estimation of live cold water coral densities.

The objective of this study is to; i) develop and compare a supervised classification of a high-resolution, reef-scale, deep-water coral image mosaic of the Piddington Mound (Porcupine Seabight, NE Atlantic) using MOBIA and a machine learning classification (MLC) to an established manual classification and; ii) establish the most appropriate machine learning classification for integrated ROV bathymetric and video datasets. In a broader context, we use the findings of this work to provide unique insights into the spatial organization of coral frameworks and environmental factors on CWC mounds. The results of this work can be used as a quantified baseline to which other coral mound surfaces can be compared. For instance, this whole reef approach would enable more accurate quantifications for carbon budget analyses and would allow time series study to assess changes in the reefs. Furthermore, the MLC applied here has a broad significance as it can easily be shared, customised and applied to other deep- and shallow-water habitats.

## Materials and Methods

### Study Site

**Figure 1:**
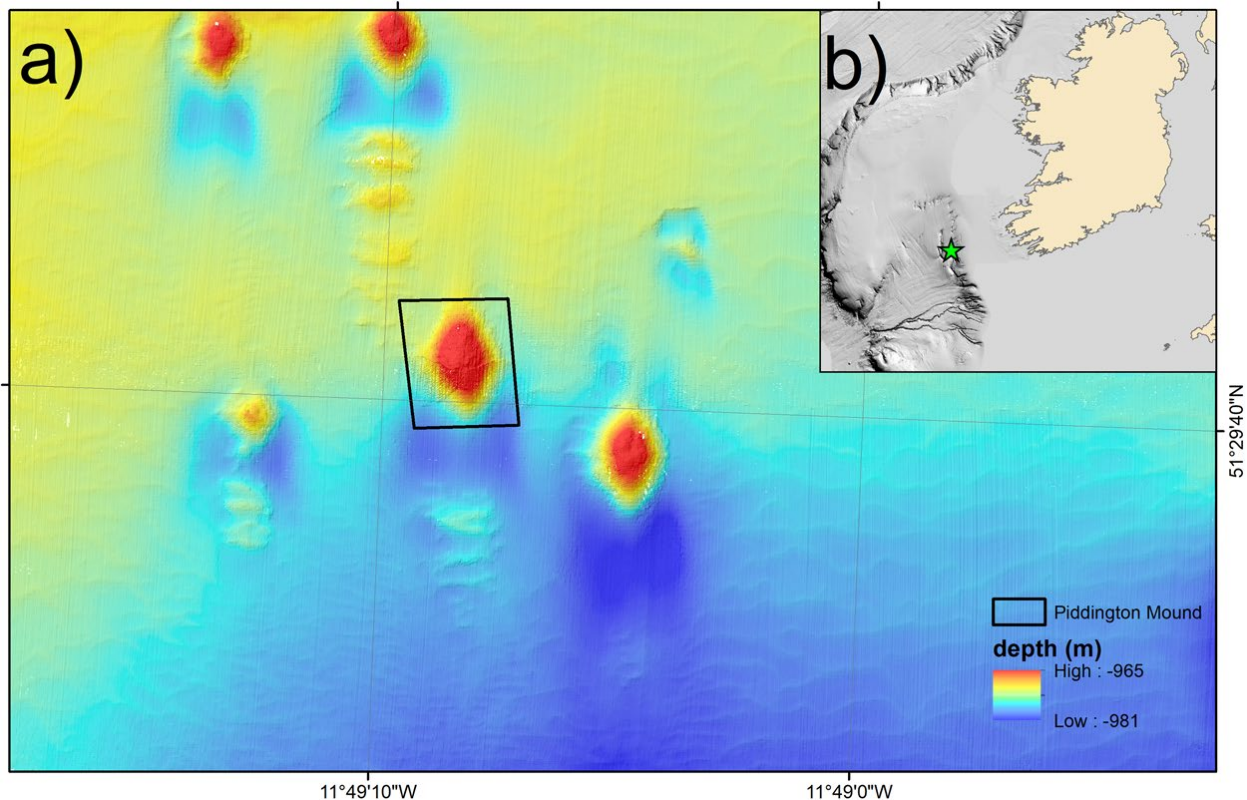
Study site area. The Piddington Mound bathymetry in the context of the Moira Mounds region derived from ROV-based multibeam echosounder data.

The Piddington Mound, a CWC mound in the Belgica Mound Province (BMP), has been selected for this study due to the existence of high-resolution imagery (video and bathymetry) which covers the entire surface of the mound as presented in^29^. The BMP is a Special Area of Conservation (SAC) located within the Porcupine Seabight, NE Atlantic (Fig. 1) that is known for the abundance of “giant” CWC mounds, up to 100 m in height, broadly aligned as two contour-parallel mound chains^13^. Smaller CWC mounds approximately 10 m tall, called the Moira Mounds, exist between these giant mounds^47,48^. The Piddington Mound is one of these smaller CWC mounds with a spatial extent of approximately 40 × 60 m and a current-aligned, ovoid morphology. Speculated to be Holocene in age^49^, the Moira Mounds are predominantly distributed across 4 areas; the northern area, the upslope area, the downslope area and the midslope area. The Piddington Mound exists in the downslope area, described as favorable for mound development with current speeds of approximately 40 cm s^−1^. Glacially-derived dropstones and fine hemipelagic sediments exist in the off-mound area, surrounding the Piddington Mound, while the mound itself is covered predominantly by *Lophelia pertusa*, *Madrepora oculata* colonies, other marine organisms such as sponges and echinoderms as well as coral rubble and sediments^29^.

### Data

ROV-video data were collected for this research during the VENTuRE survey (2011) on board *RV Celtic Explorer* with the *Holland 1 ROV* (cruise number CE11009). The video data were acquired across the Piddington Mound using a downward-facing HD camera which was mounted at the bottom of the ROV. Positioning and navigation data for the ROV during the dive were recorded using a Sonardyne Ranger 2 USBL (ultra-short baseline positioning system) corrected by an RDI Workhouse doppler velocity logger. The ROV altimeter recorded the height of the ROV (and therefore camera) above the seabed. Downward-facing HD video data were recorded during a series of transects covering the entire surface of the Piddington Mound. To maintain a clear image of the mound surface, the ROV was kept <2 m above the mound surface at a survey speed of approximately 0.8 knots. Several lights were attached to the ROV to achieve homogenous lighting across the camera field of view.

**Figure 2:**
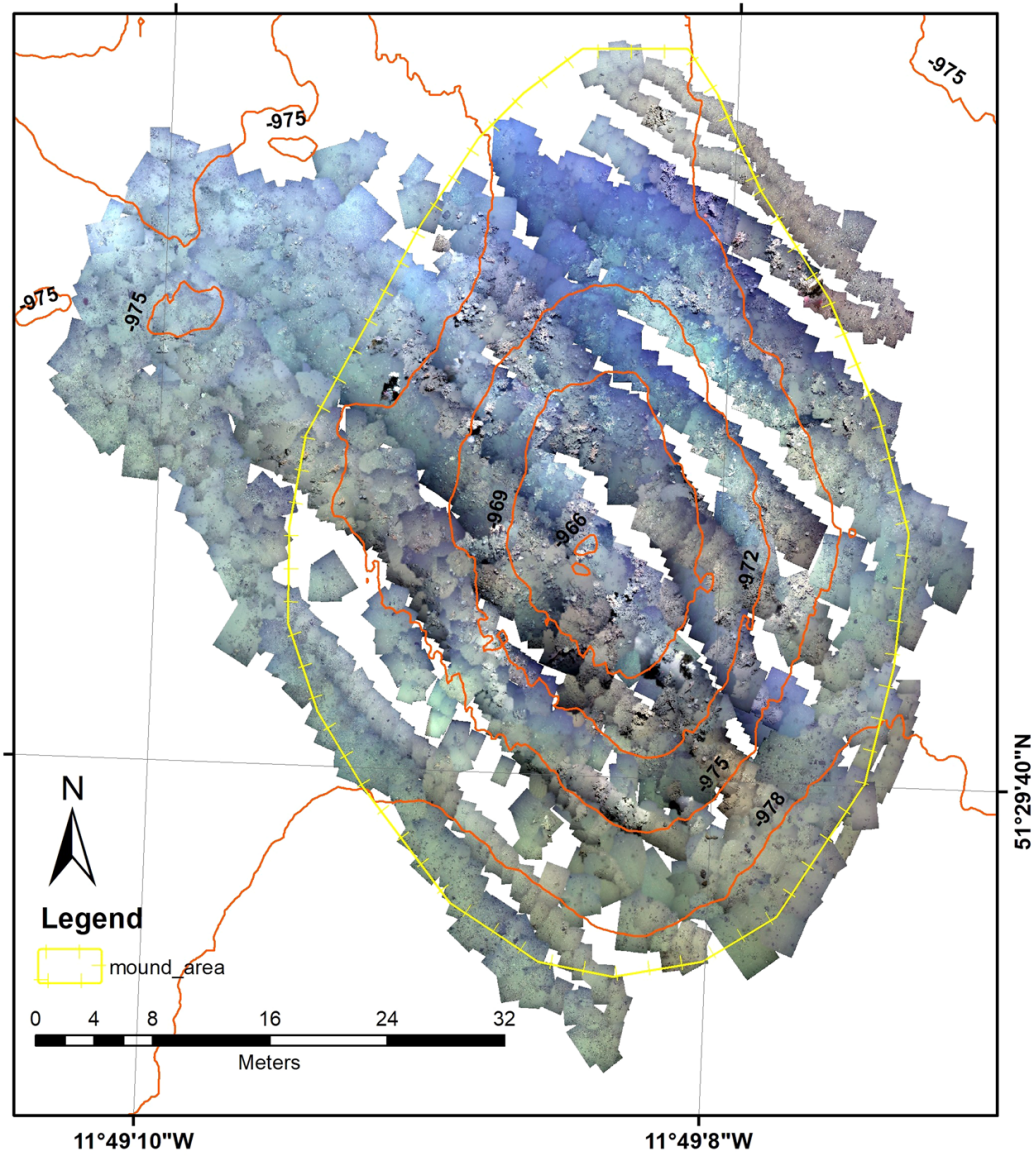
Video mosaic of the Piddington Mound. The mound area delineated by topographic break is marked by the yellow line.

The ROV-video dataset was converted into a video mosaic (Lim *et al*.^29^) using the IFREMER in-house software *Matisse* which extracted images from the raw video data at a rate of 1 per second. Poor quality video data (imagery collected more than 2 m above the seabed, collected in poor water quality, acquired too quickly or with poor navigation) were excluded from the image extraction process. To lower the trajectory noise of the ROV, sliding median filtering and 2nd order polynomial model fit was applied to all the USBL navigation data. The extracted images and filtered navigation were synchronized so that each image had an approximate position, later refined by the mosaicking process. Features in the extracted images were detected and matched using a SIFT (Scale Invariant Feature Transform) algorithm^17^. The resulting matched images and USBL navigation were merged to give an accurate global position, correct scaling and sufficient local overlapping through a cost function minimization. The final mosaic was projected within a GIS (In UTM 29N) and resampled to a 2 mm resolution. The boundary of the mound was defined by a topographical break (derived from the DTM), above which the inclination of the slope increases significantly (Fig. 2).

A Kongsberg EM2040 multibeam echosounder was mounted to the *Holland 1* ROV where it was integrated with a sound velocity sensor, Kongsberg HAIN inertial navigation system. The ROV was flown at approximately 30 m above the seabed around the Piddington Mound where multibeam data acquisition was managed and monitored via Seafloor Information System (SIS). The multibeam was operated at 400 kHz at a survey speed of 0.8 knots until data were acquired from the Piddington Mound and surrounding seabed. All data were saved as *.all files. The files were imported to Qimera where a zero tide was applied and all lines were gridded together. Using the swath editor, each line was manually inspected for anomalous data spikes, which were manually removed. The cleaned multibeam data were gridded at a resolution of 0.5 m and saved in an ArcGRID format.

### Video Mosaic Segmentation and Classification

The video mosaic segmentation process was performed to the whole video mosaic area using the multi-resolution segmentation algorithm in software eCognition v9.0^50^. Starting from an individual pixel, it consecutively merges pixels from the original until a certain threshold, defined by the scale parameter, is reached creating a polygon, or an “object” with similarities in scale/shape and brightness/colour^51^. In this way, the segmentation model parameters tuning defined by shape/colour and the scale parameter definition can be quite subjective and dependent on trial and error and the analyser’s subjectivity^52–54^. Since the main goal of this study is to perform a zonation analysis of the Piddington Mound and its main components, the optimum segmentations should be detailed enough to define units (individuals) of key biotic (e.g. coral frameworks, sponges and echinoderms) and seabed features (such as pebbles or sediment patches).

**Figure 3:**
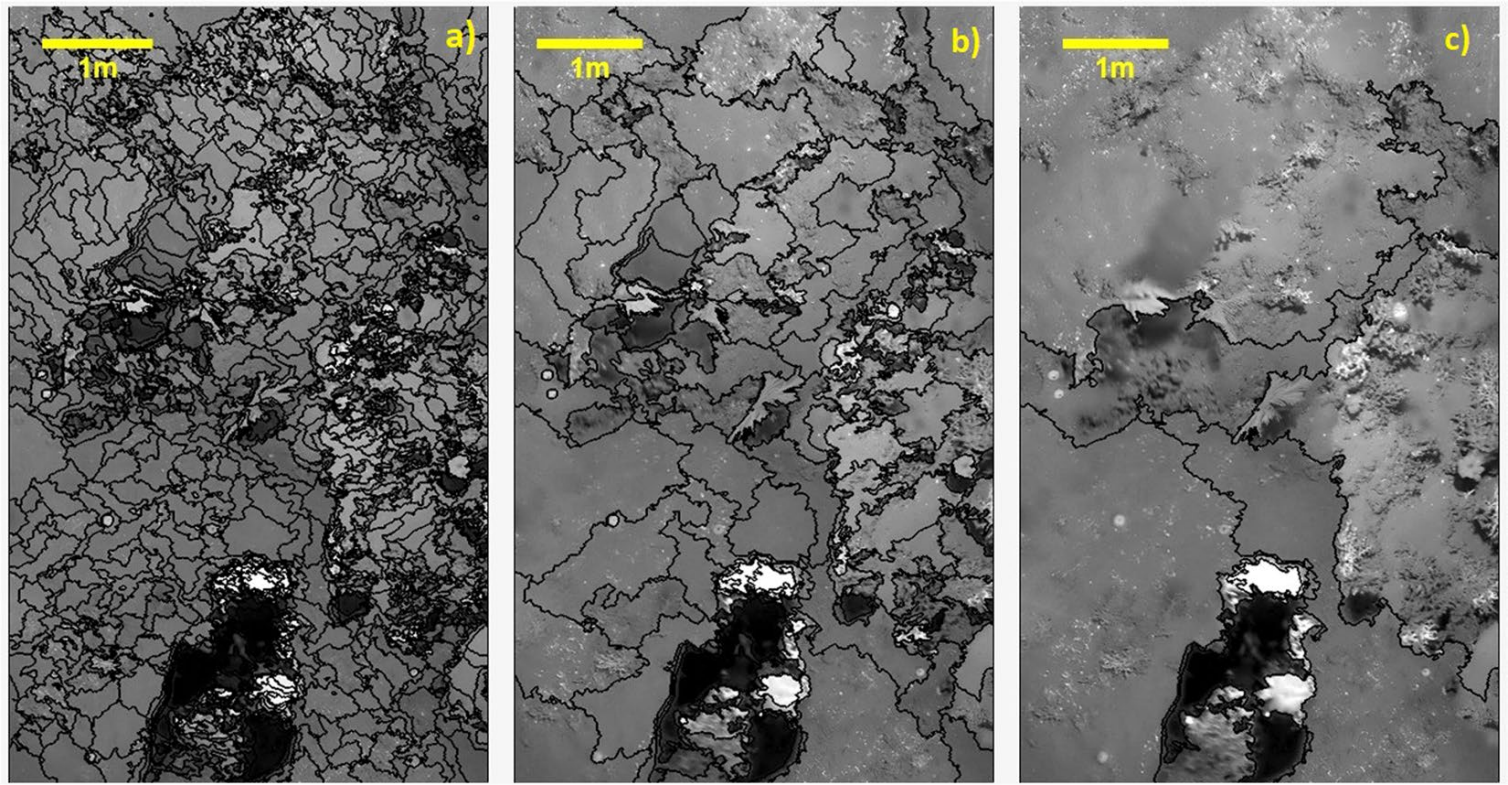
Segmentation differences for part of the study area showing examples of over segmentation. (**a**) Scale Parameter 50 (over segmentation). (**b**) Scale Parameter 400 (defined by the ESP Tool). (**c**) Scale Parameter 1000 (under segmentation).

Figure 3 shows examples of different segmentation strategies on the same areas (a central sector of the study region). Figure 3a illustrates an over-segmented section where too many objects were created. In contrast, Fig. 3c shows an under-segmented area where few objects are delineated thereby failing to reflect the bio-geophysical characteristics of the whole diversity of seabed features^55^. In order to find an optimum parameterization of the segmentation model, an Estimation Scale Parameter model (ESP), developed by^56^ was used which defines the empirical relationship between spatial structures of the image and the size of created objects. Based on the calculated results of the ESP model for the area, the Scale Parameter was defined as “400” producing an apparently adequate segmentation (i.e. with the features such as coral frameworks and sponges’ polygons were clearly delimited - Fig. 3b).

For each polygon (object) defined by the segmentation tool, a set of features were calculated relating to shape, form, texture and context. At this phase, bathymetric data were also incorporated to the dataset. Table 1 presents the details of the selected features. Detailed calculations of these texture measures can be found in^52^.

**Table 1:** Spatial features used as parameters in classification process.

Feature category	Feature type	Description
Bathymetry	Depth, slope, aspect and curvature	Values of mean values of depth, 1st and 2nd derivative (slope and curvature) and orientation (aspect) – derived from multibeam bathymetry
Optic	Mean red, mean green, mean blue, brightness	Mean values of light intensity in each RGB channel and total brightness (sum of the object means in all bands). – derived from video mosaic imaging
Texture	Homogeneity, entropy, mean, backscatter	Texture features are derived from texture after Haralick based on the Gray-Level Co-occurrence Matrix or Gray-Level Difference Vector. derived from multibeam backscatter.
Shape	Area, compactness, density, roundness, main direction, rectangular fit, elliptic fit, asymmetry, border index, shape index	Shape features refer to the geometry and information of the segment objects. derived from a multi-resolution segmentation algorithm

**Figure 4:**
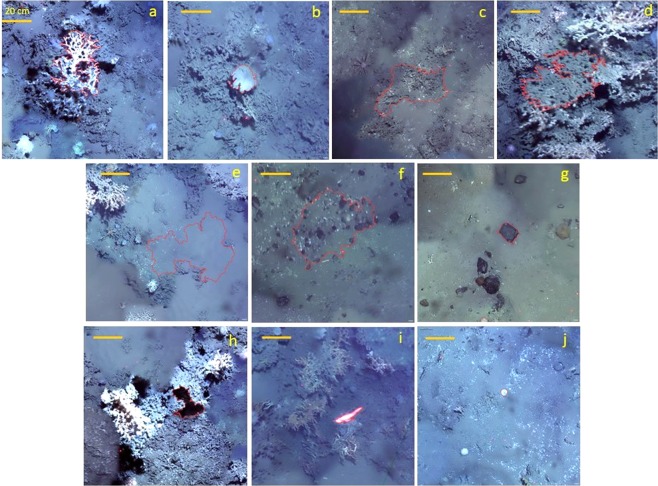
Segments (objects) based on self-existent and resoluble entities: biogenic divided into (**a**) living coral framework (mainly *Lophelia pertusa* and *Madrepora oculata*); (**b**) glass sponges; (**c**) coral rubble and; (**d**) dead coral framework. Sediment divided into (**e**) hemipelagic sediments; (**f**) hemipelagic sediment with dropstones and; (**g**) pebbles. Non-classified divided into (**h**) shadows; (**i**) fish and; (**j**) echinoderm/others. The yellow bar represents a 20 cm scale reference.

The next step was the classification process which assigns map categories to each segment using membership rules^57^. It defined hierarchical class labels based on three distinct categories: (1) Biogenic; (2) Sediment and; (3) other/non classified. The Biogenic category was subdivided in “live coral framework”, “dead coral framework” and “sponges”. The Sediment category was subdivided in “hemipelagic sediment” (sand or mud with no recognisable bioclasts or dropstones), “hemipelagic sediment with dropstones” and “dropstones/pebbles”. The category “other/non classified” was related to objects with few representatives in the overall environment (e.g. fish, crab, echinoderms) or with no significance (e.g. shadows). The categories were based on the work of^29^ in the same area (the Piddington Mound). Figure 4 shows examples of selected categorical objects of each class label.

150 training samples were selected for each significance category except the “*other*” category which was not considered for this analysis due to its rare occurrence and insignificance in the area as a whole. Three different supervised classification methods were applied: Log Regression (LR), Random Forest (RF) and Deep Neural Network (DNN). The training and classification models were developed in Microsoft Azure Machine Learning Studio (MAMLS)^58^ and implemented in Python 3.4.

LR Multiclass supervised classification is an extension of binary logistic regression which categorizes objects based on their closest training samples in feature space predicting class probability based on the input features after ranking them according to their relative importance^59–61^. Random Forest is a method that operates by constructing multiple decision trees (i.e. classification trees, where the leaves represent classifications and the branches represent conjunctions of features that produce those classifications). Then by voting for the highest output class, it searches away from the unknown object to be classified in all directions until it encounters *k* user-specified training objects and assigns the object to the class with the majority vote of the encountered objects^62,63^. The DNN method is inspired by the way biological nervous systems process information. It consists of a set of interconnected layers, in which the inputs lead to outputs by a series of weighted elements (edges and nodes of Neural Networks). A particular set of neural network algorithms, made up of more than three layers along with the input, output and more than one hidden layer, are known as “Deep” learning algorithms with the method itself is referred to as a “Deep Neural Network”^64,65^.

**Figure 5:**
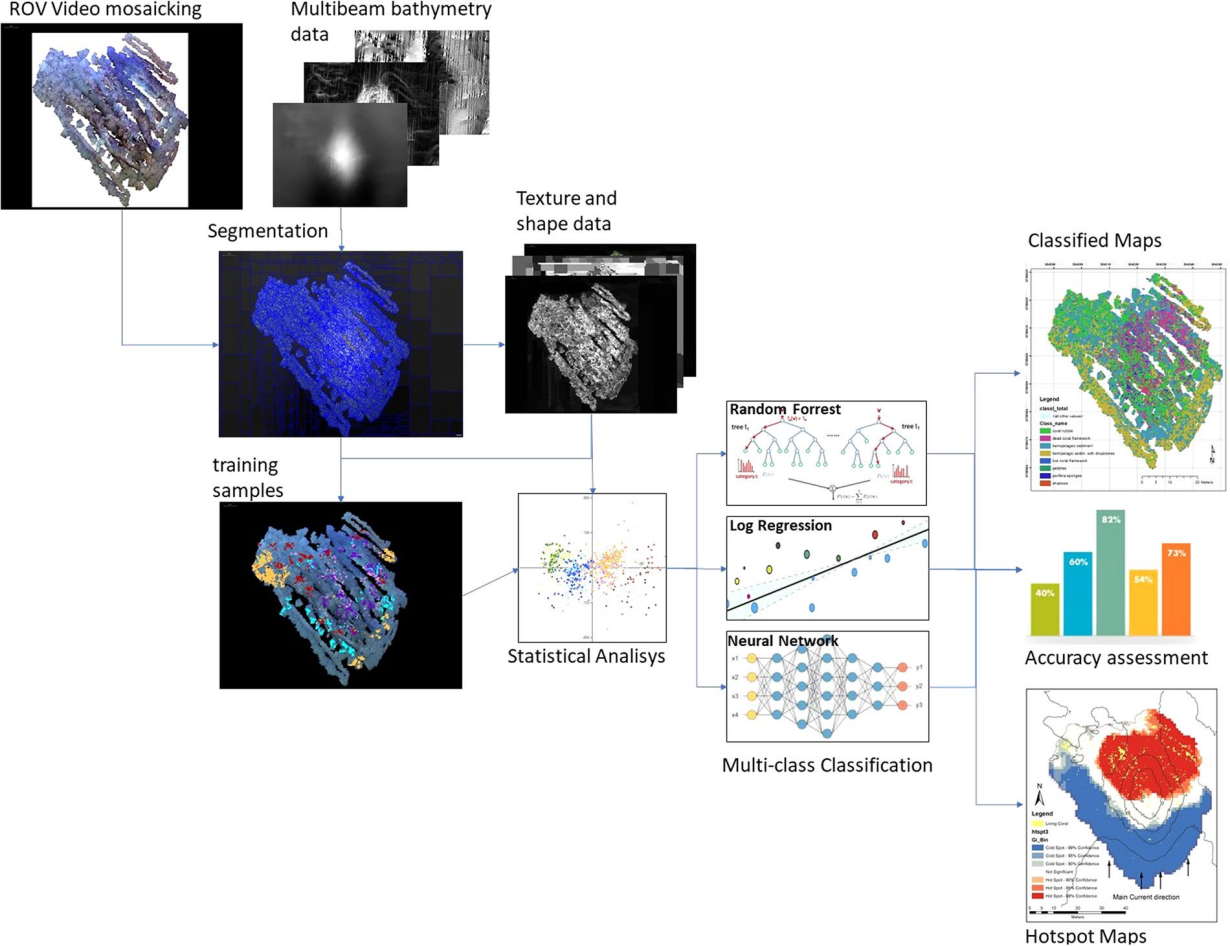
Methodology workflow proposed for the Piddington Mound classification.

As the living biogenic facies (i.e. living corals and sponge objects) covers quite a small individual area in relation to the total area, it can be difficult to characterize the distribution of these classes in the context of the mound. In order to better describe the spatial distribution of these classes, “hot spot” analysis using Getis-Ord GI* function was performed. This tool works by looking at each feature within the context of neighboring features (see^66,67^). For all methods, classification model frameworks were built based on data preparation, training, model creation using DNN, RT and LR algorithms and model accuracy evaluation. The output classification results were exported into a geodatabase for subsequent spatial analysis and map preparation (see Fig. 5).

## Results

### Accuracy assessment

**Figure 6:**
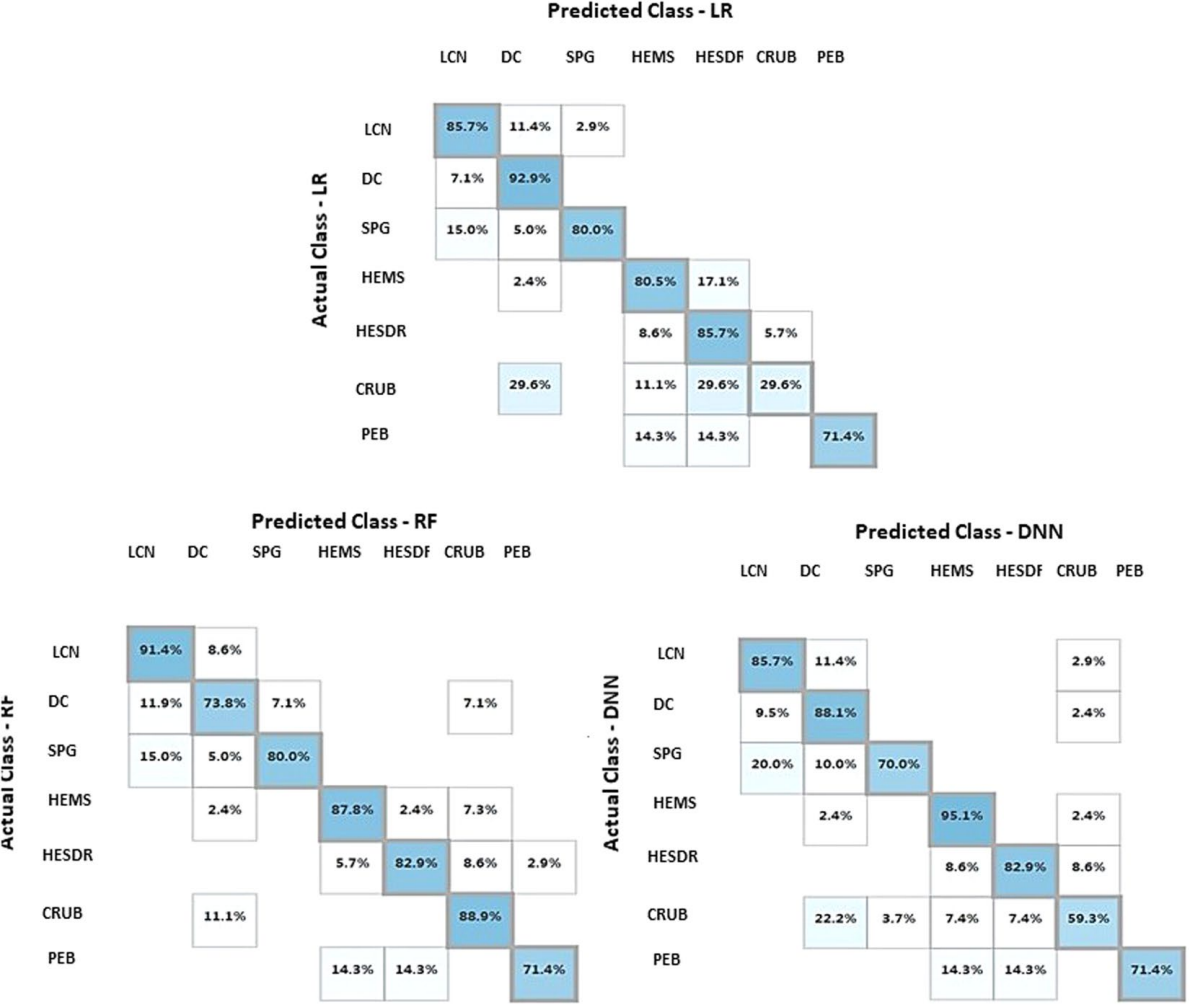
Accuracy matrix for classification methods LR, RF and DNN: Classes:LCN) Live Coral Framework; DC) Dead Coral Framework; SPG) Sponges; HEMS Hemipelagic Sediments; HESDR) Hemipelagic Sediments with Dropstones; CRUB) Coral Rubble; and PEB) Pebbles.

For the analysis of class prediction, a final accuracy assessment of 15% of the dataset were sampled randomly for training and the remaining 85% were used as test set. The multi-class classification performance matrix taken from MAMLS model set is shown in Fig. 6. The precision and recall matrices were assessed by two different metrics: micro-averaging and macro-averaging. Micro-averaging tends to be effective in the most frequent classes whereas macro-averaging considers each class equally. The experimental result of the models are presented in the Table 2.

**Table 2:** Accuracy metrics for the classification methods.

Metric	LR	RF	DNN
Overall accuracy	0,7778	0,8213	0,8357
Average accuracy	0,9365	0,9489	0,9531
Micro-averaged precision	0,7778	0,8213	0,8357
Macro-averaged precision	0,8268	0,8519	0,8366
Micro-averaged recall	0,7778	0,8213	0,8357
Macro-averaged recall	0,7512	0,7893	0,8232

### Classification

**Figure 7:**
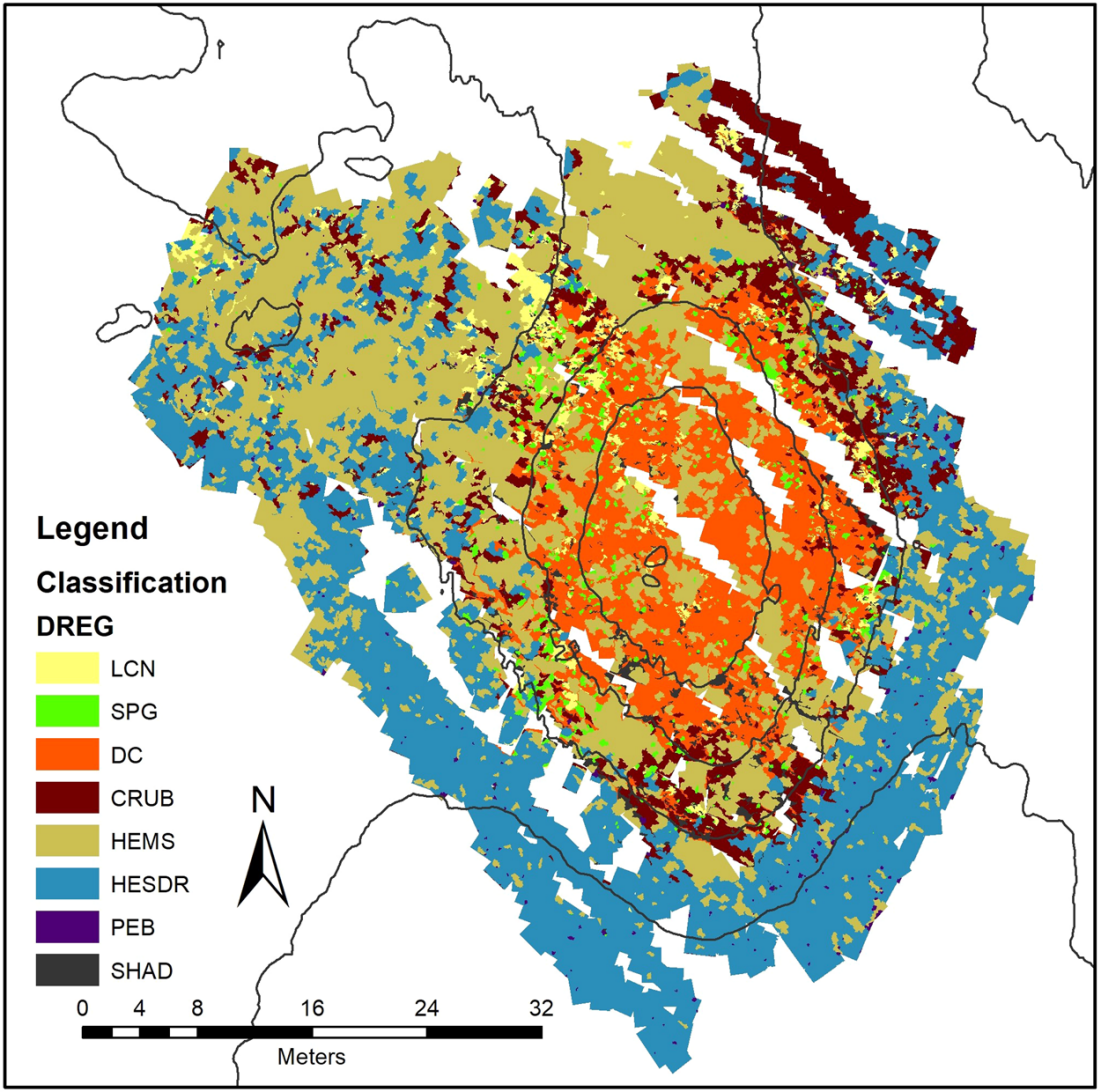
Classified Map - Log Regression (LCN) Live Coral Framework; (DC) Dead Coral Framework; (SPG) Sponges; (HEMS) Hemipelagic Sediments; (HESDR) Hemipelagic Sediments with Dropstones; (CRUB) Coral Rubble; (PEB) Pebbles and (SHAD) Other/Shadows.

**Figure 8:**
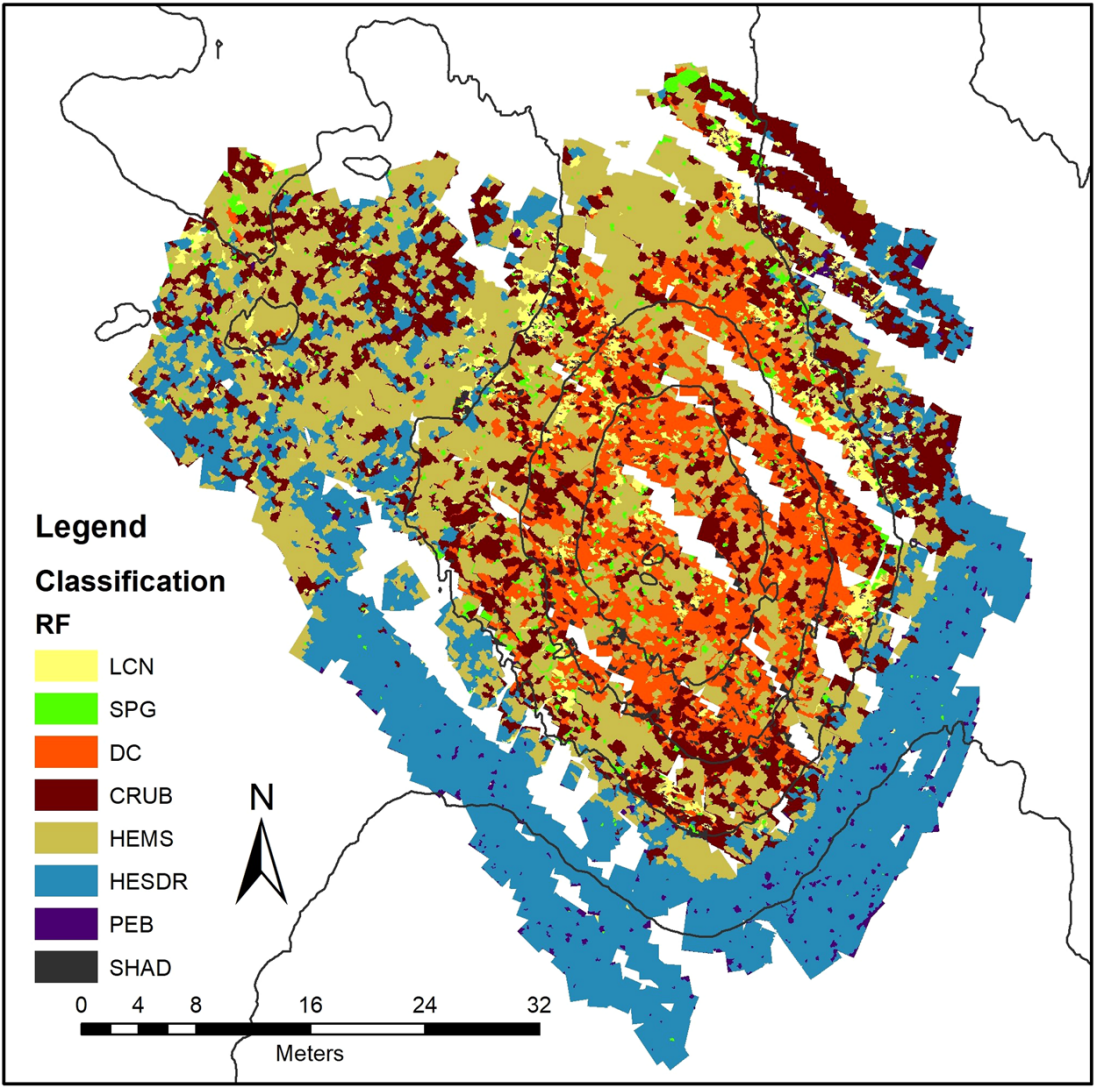
Classified Map - Random Forrest (LCN) Live Coral Framework; (DC) Dead Coral Framework; (SPG) Sponges; (HEMS) Hemipelagic Sediments; (HESDR) Hemipelagic Sediments with Dropstones; (CRUB) Coral Rubble; (PEB) Pebbles and (SHAD) Other/Shadows.

**Figure 9:**
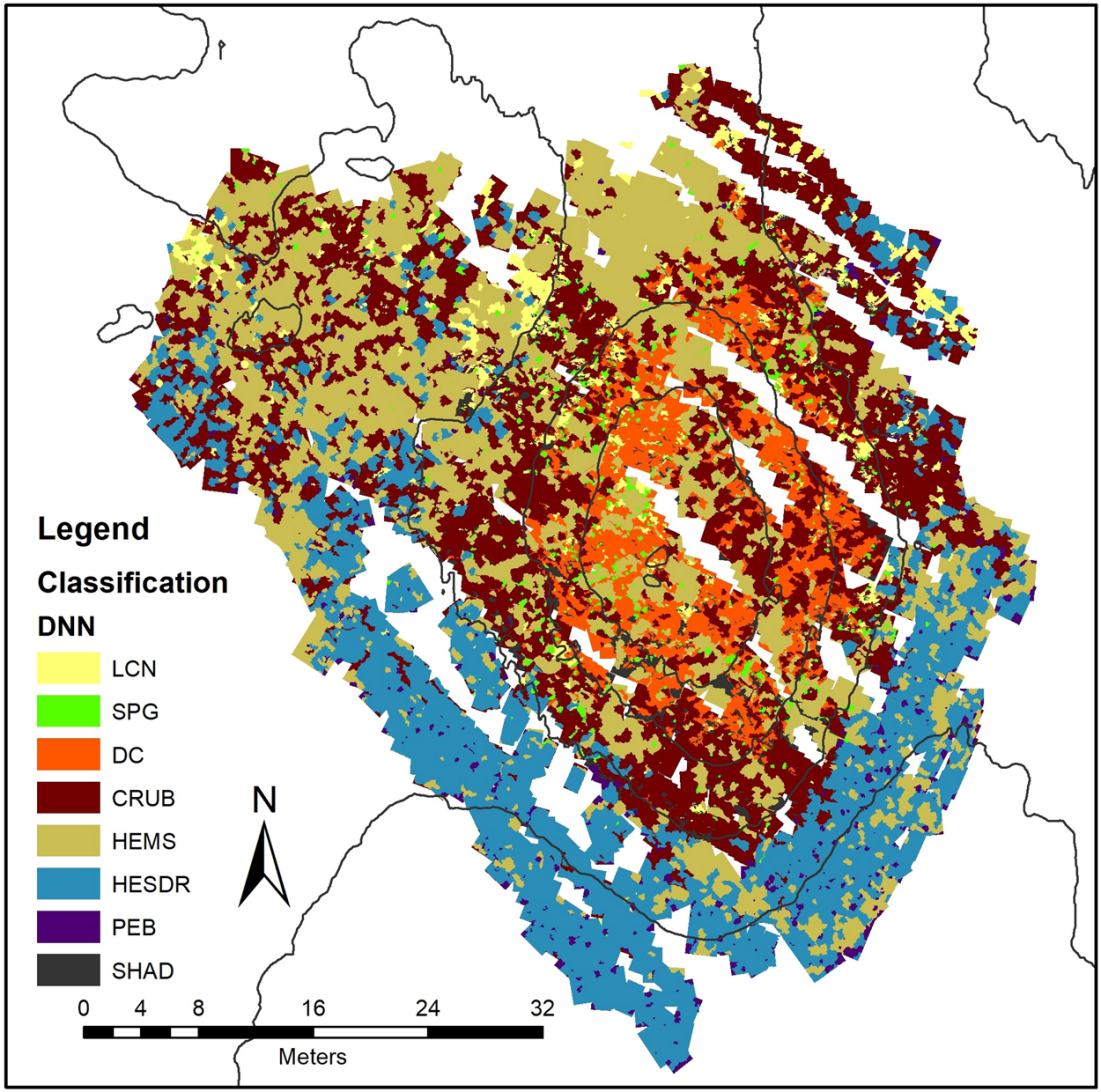
Classified Map – Deep Neural Network (LCN) Live Coral Framework; (DC) Dead Coral Framework; (SPG) Sponges; (HEMS) Hemipelagic Sediments; (HESDR) Hemipelagic Sediments with Dropstones; (CRUB) Coral Rubble; (PEB) Pebbles and (SHAD) Other/Shadows.

The classified maps generated by the three classification models are presented in Figs 7, 8 and 9 and the area and object counting summary is presented in Table 3. The general trend of the class distribution is quite similar in the three methods, with overall accuracies between 87% and 83% (Table 2). Although the RF and LR classifiers yielded the lowest general results, they allow the interpretation and control of their parameters with adjustments and rules settings. In contrast, DNN are more complex where interpretation is more difficult and can only be verified externally^37^.

**Table 3:** Areas and object statistics – % T.A. (% of object in total area), # of Obj. (total number of objects) M.O.A. (mean size of each object, in m^2^) % M.A. (% of area inside the mound limits).

	total area	% T.A.	# of obj.	M.O.A.	area mound	% M.A.
**DNN**						
coral rubble	676,12	28,98	3680	0,18	474,37	29,89
dead coral	199,38	8,54	1641	0,12	199,37	12,56
hemim. Sediments	703,39	30,14	3197	0,22	433,87	27,34
hem. sed. with drops	549,40	23,54	2000	0,27	324,01	20,42
sponges	46,22	1,98	739	0,04	56,62	3,57
live coral	81,94	3,51	1041	0,07	32,38	2,04
other	76,98	3,30	1630	0,04	66,21	4,17
	2333,43	100,00	13928	0,16	1586,83	100,00
**LR**						
coral rubble	235,67	10,10	1567	0,15	169,24	10,67
dead coral	354,44	15,19	2521	0,14	354,44	22,34
hemim. Sediments	809,45	34,69	3952	0,20	506,74	31,93
hem. sed. with drops	767,45	32,89	2841	0,27	410,05	25,84
sponges	44,67	1,91	824	0,05	43,34	2,73
live coral	69,36	2,97	1105	0,06	57,94	3,65
other	52,39	2,25	118	0,04	45,08	2,84
	2333,43	100,00	13928	0,16	1586,83	100,00
**RF**						
coral rubble	449,11	19,25	2273	0,19	297,74	18,76
dead coral	300,98	12,90	2224	0,13	299,24	18,86
hemim. Sediments	693,42	29,72	3187	0,21	460,60	29,03
hem. sed. with drops	696,60	29,85	2734	0,25	369,75	23,30
sponges	44,41	1,90	1308	0,07	36,96	2,33
live coral	94,01	4,03	852	0,05	69,52	4,38
other	54,90	2,35	1350	0,03	53,02	3,34
	2333,43	100,00	13928	0,16	1586,83	100,00

For the “sediment cover” classes (i.e. “Coral Rubble” and “Hemipelagic sediments”), the accuracy analysis (Fig. 6) shows a significative drop in statistical confidence especially in LR and RF methods for both classes. The DNN method however, maintained an accuracy higher than 80% for these classes indicating a better discrimination capability between such classes. Visual inspection confirmed that the results of the DNN were more effective, although in some cases it can be difficult to differentiate between classes even visually.

For smaller and more distinct objects such as sponges and living corals, the results were less uniform with an accuracy of >80% (except in RF classification for sponges which had a confidence of approx. 70% where the classification has an issue discerning between living and dead coral framework).

The zonation trend of the mound is quite clear with a prevalence of hemipelagic sediment with dropstones and larger pebbles on the deeper, southern part of the mound while at the northern, marginal areas the classes “hemipelagic sediment” and “coral rubble” are more dominant. The mound itself is covered by biogenic facies “dead coral framework” and “coral rubble”. At the highest parts of the mound (below −974 m depth), both classes represent more than 60% of the coverage (against approx. 36% in overall area).

**Figure 10:**
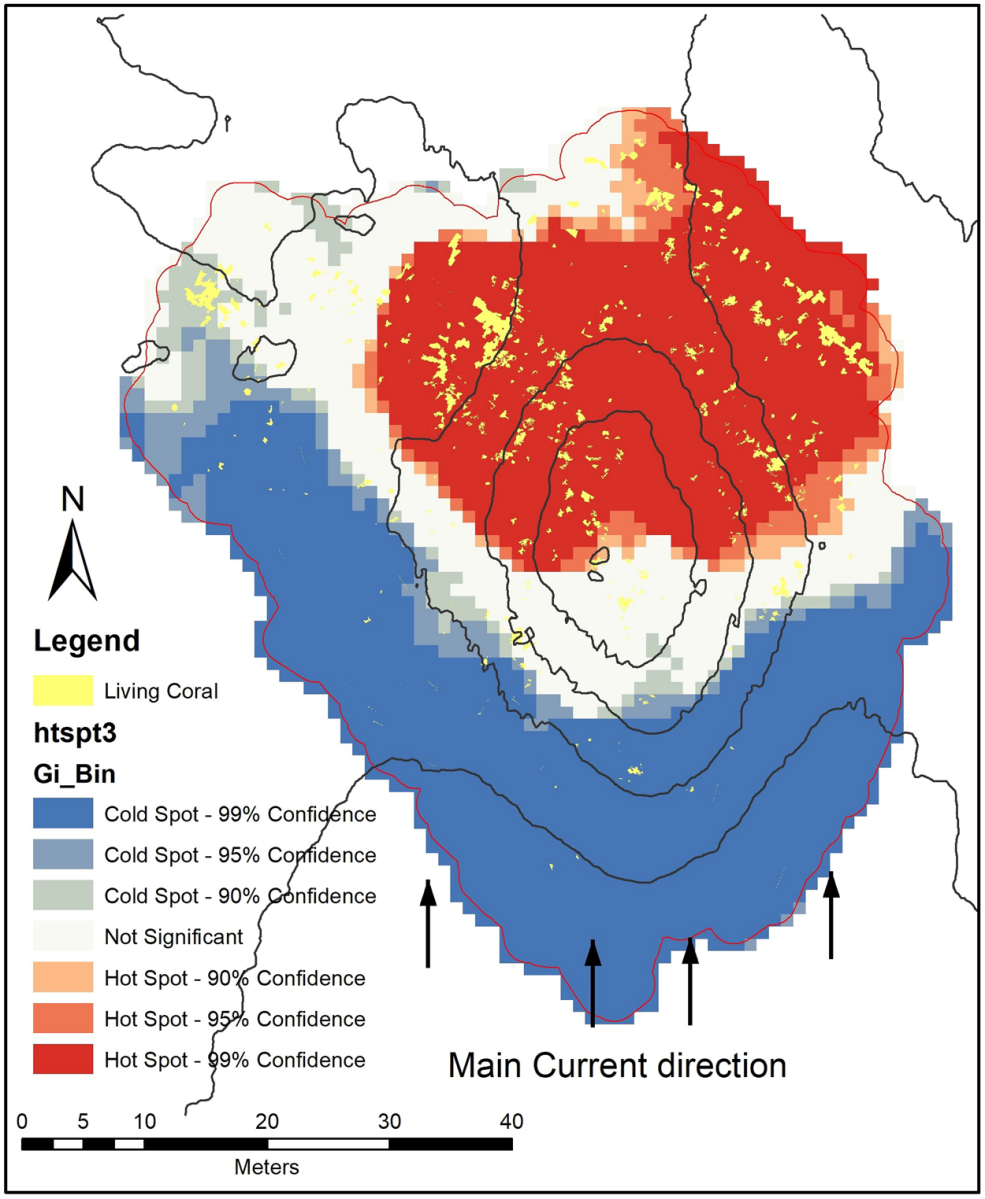
Hot Spot map of coral object distribution.

**Figure 11:**
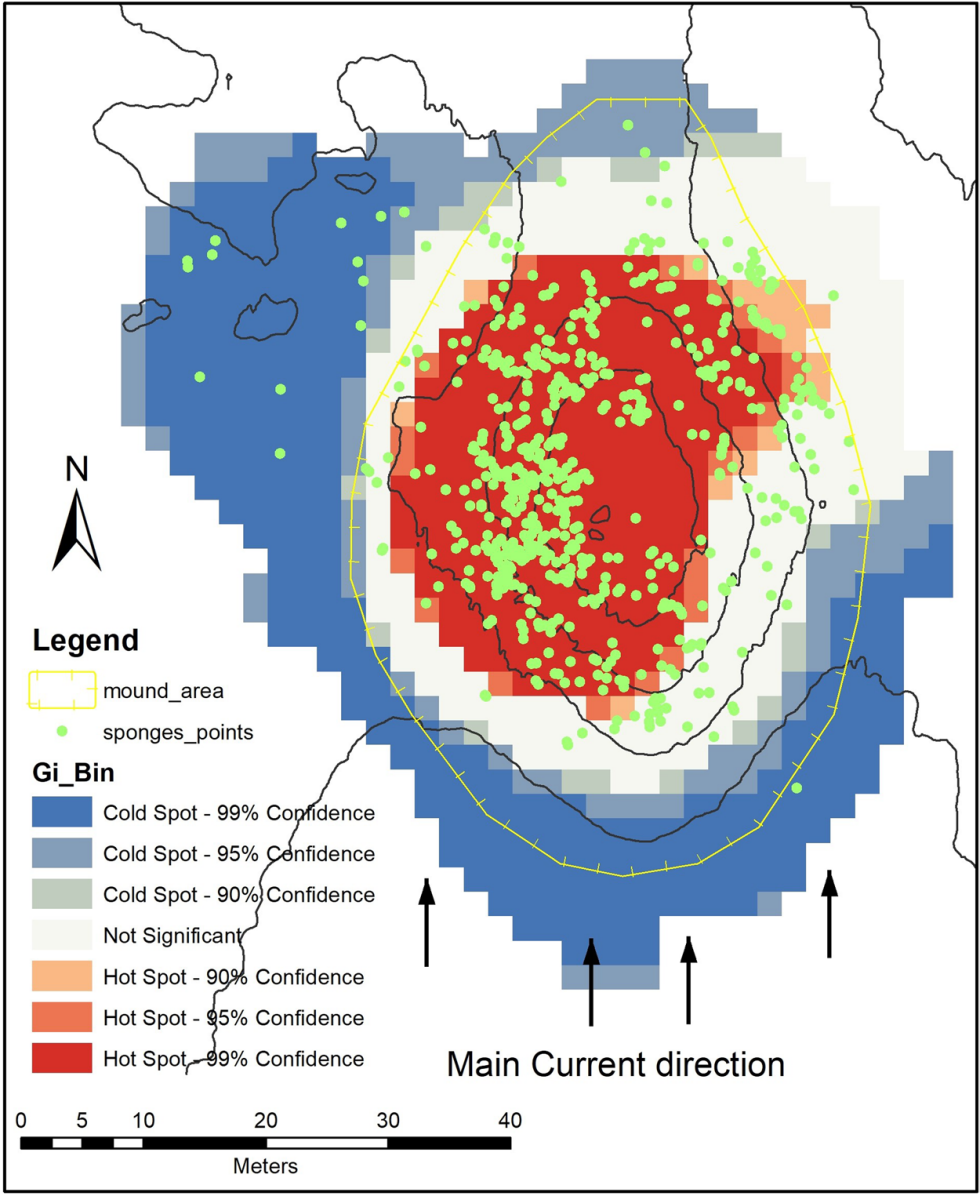
Hot Spot Map of sponges’ object distribution.

**Figure 12:**
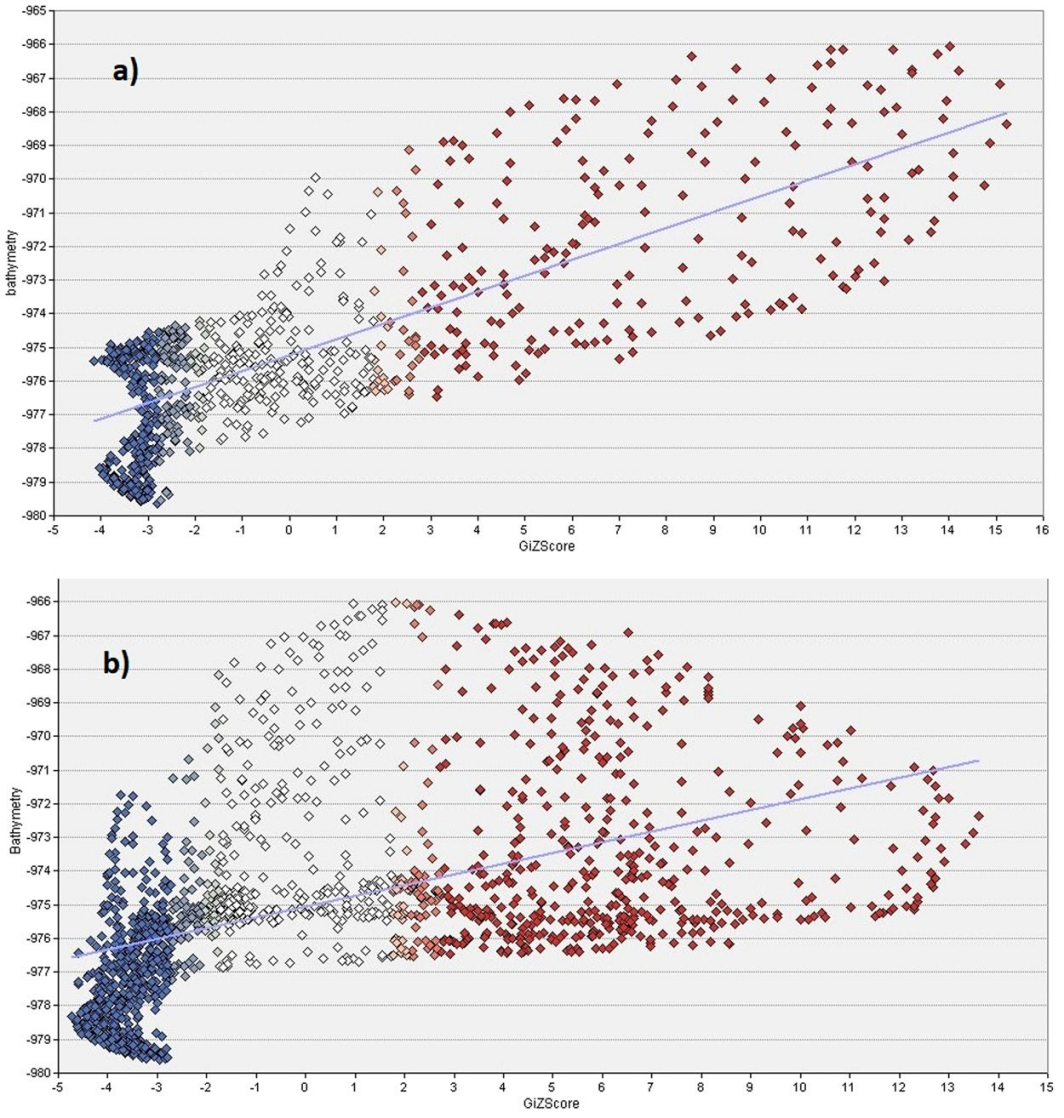
Scatter plot for between Z-score of glass sponges (**a**) and coral (**b**) hotspot cells (with colour scale related to Figs 10 and 11) and the bathymetry.

Living biogenic facies (i.e. living corals and sponge objects) have a relatively small individual area and can be difficult to identify in the general map context. As such, the “hot spot” map (Fig. 10) shows that high concentrations of living corals are located to the north face of the mound, with very few occurrences outside the 99% confidence area. Glass sponges clearly occur and are related to both the mound summit and the upper western face of the mound (Fig. 11) (note, the sponges class is represented by dots due to the small dimensions of polygons). The scatter-plot graphs (Fig. 12) show a clear correlation between Z-score of hotspot cells and the bathymetry of sponges (a) indicating a high correlation of sponge occurrence and topographical settings. while the trend is much less evident among corals (12 b).

**Figure 13:**
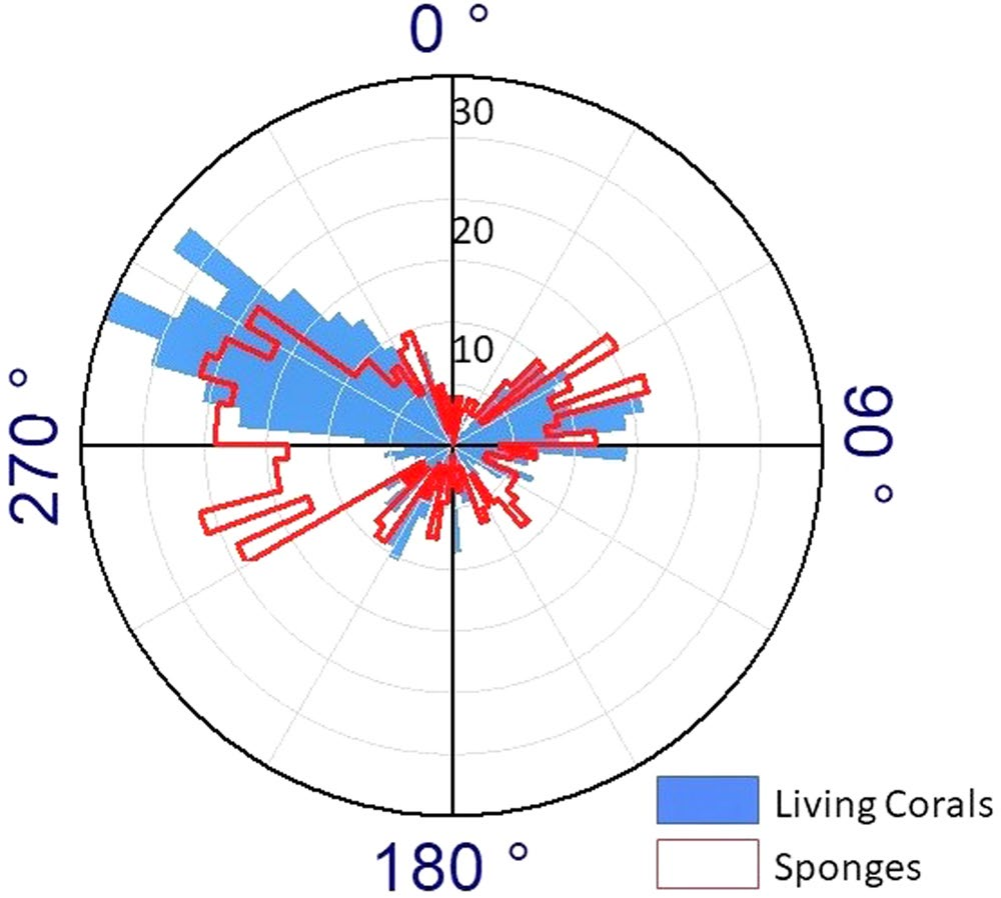
Polar plot indicating mound slope orientation where coral and sponges have settled.

The polar plot of the Living coral and Sponges (Fig. 13) shows the distribution of living corals and sponges in relation to slope orientation or aspect. It is very clear that the arrangement of living organisms (sponges and corals) on the mound obeys a distribution pattern restricted to the northern mound flank. In the case of corals, there are two oppositely symmetrical directions: approximately 300° (WNW) and 70° (ENE). A similar but less tight relationship between occurrence and aspect is also revealed for sponges.

## Discussion

The quality and accuracy of object-based image analysis applied to CWC mound photo mosaicking as described in this paper are dependent on two steps: Segmentation and Machine learning classification algorithms. The segmentation process is dependent on empirical calibration, the basis of a trial and error procedure where the optimum level of feature discrimination can be reached. New methods of Object-based scale parameter selection have been proposed and applied^38,68,69^ but Kim *et al*.^70^ noted that defining the most suitable scale for image segmentation is still quite problematic. In this case, we applied a local variance (LV) of object heterogeneity to determine the best scale parametrization as proposed in^56^. However, in some cases, areas with sponges, pebbles and smaller objects such as isolated corals and echinoderms were under-segmented when they existed over complex backgrounds (such as coral rubble or dead coral framework). The diminution of scale parameter, yielding the creation of detailed segments, resulted in a considerable increase in processing time and over-segmentation of objects which decreased the accuracy of the classification algorithms.

The Machine learning classification models have produced satisfactory results by creating facies distribution maps of the Piddington mound with improved results compared to the manual counting method described by Lim *et al*.^29^. Some advantages of the ML object classification can be highlighted: (1) processing and modelling time is considerably lower especially after tuning the segmentation and classification algorithm parameters; (2) it offers a less-subjective and observer-dependent approach to mapping and classifying coral facies; (3) it can be specific to typical coral facies quantification and characterization (i.e. sponges, coral framework) by tuning the parameters to better perform identification (segmentation) and classification of these features. Subsequently, the algorithm can be trained to compare different sites and therefore indicate quantitative structural differences between frameworks (i.e. mound, reefs); (4) it produces an ordinary georeferenced file in a geodatabase format (e.g. “shapefile”) that can aggregate more information into object fields such as taxonomic or sampling data which can increase the accuracy of new classification processes and; (5) it allows the manipulation of classes (signing, recombining, merging).

In terms of overall classification, all three methods tested herein produced similar results to map mound facies. For living organism classes (living coral and sponges) the DNN method showed a better discrimination performance in terms of accuracy level as well as with a lower misclassification error (mainly related to differentiation between living and dead coral framework). There was a relevant classification confusion between sponges and living corals in all methods. Visual inspection of these misclassified objects indicates that since sponges almost always appear in isolated occurrences (and the samples were chosen in this condition), when they occurred in pairs or more adjacent individuals, the models invariably classify them as “living corals”. This issue can be addressed by “re-segmentation” of the area with a lower scale parameter. This would help to identify individual organisms as smaller segments (objects) however, it is important to note that this may lead to a lower classification performance in other classes.

In the case of non-living classes, the major problems of classification occurred in the differentiation of allogenic and autogenic carbonate-dominated substrates (coral rubble and dead coral framework) (see Fig. 6). The DNN method was able to differentiate between these classes which can be evidenced at the top of the mound where there are significantly more “rubble coral” classes in the DNN model map reflected by a higher accuracy for this class (88% against 59.3% and 29.6% for RF and LR, respectively). For “sediment substrates” (Hemipelagic Sediments and Hemipelagic Sediments with dropstones) the methods also showed similar results with good discrimination rates and accuracy (>80%).

The MOBIA carried out here offers an accurate quantification of the amount of Coral Framework (12%; ~3.5% live and ~8.5% dead) and sponges (2%) across the Piddington Mound. This is the first object-level estimation of live and dead coral framework facies and individual sponges across an entire CWC mound. Interestingly ~29% of the mound surface is covered by coral rubble.

While coral rubble and dead coral framework are found across the mound, the live coral framework is restricted to the northern sector of the mound. The distribution of sponges also shows a hot spots distribution towards the highest parts of the mound, such pattern was not observed with the living corals (Fig. 12a,b). Lim *et al*.^29^, suggest that this restriction can be attributed to the high current speeds in the area and the relation to optimal conditions for coral feeding and hydrodynamics and for larvae to find hard substrates to attach to^71^.

Given the northern restriction of the live coral frameworks, a continued development of the mound in contemporary conditions is likely to generate an asymmetric north-south mound profile. Conversely, the mound has a north-south symmetric elongation (Fig. 1). As such, it is unlikely that the distribution of the live coral has been restricted to the northern portion of the mound throughout its development. Furthermore, given the occurrence of dead coral framework across the full mound which has not yet been buried by sediment or bioeroded due to long exposure, this suggests that the northern restriction is related to a recent change in environmental conditions. This suggestion can also be strengthened by recent observations which show that there has been a total of 19% change in the proportion of sediment (hemipelagic and bioclastic) and coral frameworks (live and dead) on the Piddington Mound surface from 2011 to 2015^22^.

## Conclusion

Three different machine learning classification methods (decision tree, logistic regression, and multilayer deep neural network) were applied to a high-resolution, segmented, reef-scale video mosaic and ROV-mounted multibeam data. The results show that Object-Based Image Analysis (OBIA) derived from the grouping of similar pixels in “objects” (self-existent and resoluble entities) with similar characteristics was particularly successful when applied to high resolution marine habitat mapping, specifically to cold water coral mound facies. Further, the concept of “MOBIA” (Marine OBIA) might be used since it considers not only optical imaging, but also acoustic data to segment and classify seabed features. Such techniques proved considerably more effective than manual and/or pixel-based approaches.

In order to perform MOBIA, segmentation and classification methods should be applied. Although some models for estimating segmentation parameters exist, the process of defining optimal characteristics of objects (according to seabed variables) is still largely empirical and analyst-dependent. New Machine Learning classification methods, widely available from private and open source platforms, has proven to be quite successful in terms of mapping accuracy. Deep Neural Networks showed an overall higher classification accuracy, although the Random Forest and Log Regression showed similar results.

Given the spatial coverage (100% of the CWC mound) and data resolution (2 mm video mosaic and 10 cm bathymetry), MOBIA was applied to an entire CWC mound for the first time to quantify individual organisms (e.g. sponges), coral framework coverage and typical sedimentary facies. The results show that the mound has a high coverage of coral rubble (29%) and only 12% of the mound was covered by coral framework.
